# Repurposing phosphodiesterase-5 inhibitors as chemoadjuvants

**DOI:** 10.3389/fphar.2013.00082

**Published:** 2013-06-25

**Authors:** Amit K. Tiwari, Zhe-Sheng Chen

**Affiliations:** ^1^Department of Biomedical Sciences, College of Veterinary Medicine, Nursing and Allied Health, Tuskegee UniversityTuskegee, AL, USA; ^2^Department of Pharmaceutical Sciences, College of Pharmacy and Health Sciences, St. John's UniversityQueens, NY, USA

Phosphodiesterase-5 (PDE5) inhibitors have shown a beneficial effect in a variety of clinical conditions, such as benign prostate hyperplasia, pulmonary arterial hypertension, female sexual arousal disorder, overactive bladder, and incontinence, Raynaud's disease, heart failure and stroke among others (Sandner et al., 2007). Three PDE5 inhibitors, sildenafil (Viagra™), tadalafil (Cialis™) and vardenafil (Levitra™) are clinically approved and are widely used for the treatment of erectile dysfunction. A retrospective analysis determined that men treated with PDE5 for ED had less chance of having prostate cancer. This population of men had significantly lower documented diagnosis of elevated prostate-specific antigen and higher percentage of benign prostatic hyperplasia compared to men not treated with PDE-5 inhibitors (Chavez et al., 2013). Emerging evidence indicates that PDE5 inhibitors are multi-targeting agents and have promising results in the treatment of variety of tumors. Here we propose the possibility of repurposing of PDE5 inhibitors for adjuvant chemotherapy.

**Figure 1:**
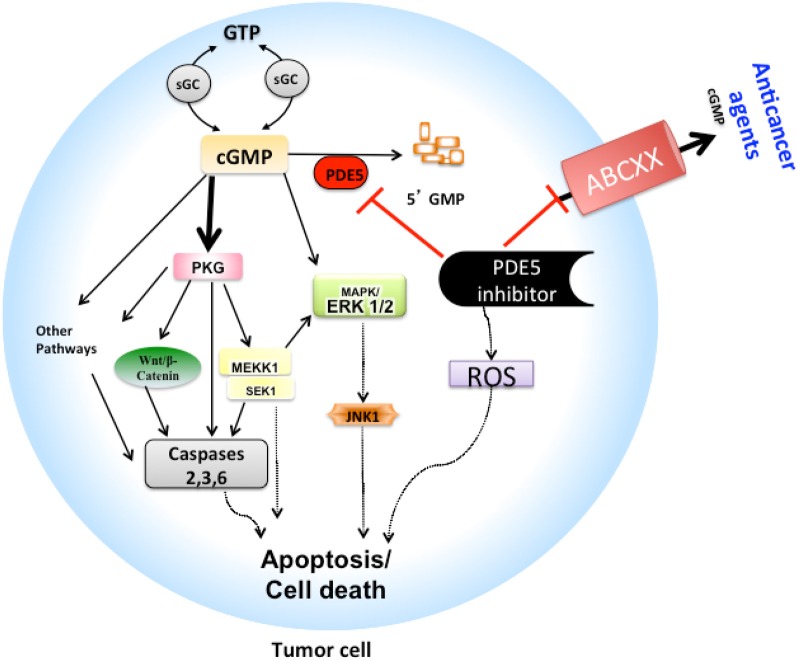
**Schematic model of PDE5 inhibitors mechanism as chemoadjuvants: Proposed model is shown (A) as how tumor cells can efflux cGMP and variety of anticancer drugs that are substrate of ABCB1, ABCG2, ABCC4, ABCC5, and ABCC10 and so survive in the absence of PDE5 inhibitors. (B)** Probable mechanism by which the PDE5 inhibitors such as sildenafil, vardenafil and tadalafil produces apoptotic activity is shown, which may be due to (1) inhibition of PDE5 activity, thus increasing cGMP-PKG activation, which leads to activation of series of signaling events including phosphorylation of β-catenin and/or MEKK1/SEK1/JNK1 signaling pathways that eventually results in apoptosis cascade, and/or (2) inhibition of efflux function of ABCC4, ABCC5, ABCC10, ABCB1, and ABCG2 drug transporters and thus increase the sensitivity of other chemotherapeutic agents that are substrates of these transporters. ABCXX, refers to ABCB1, ABCG2, ABCC4, ABCC5, or ABCC10; cGMP, cyclic guanosine monophosphate; ERK1/2, extra-cellular regulated kinases 1/2; MAPK, mitogen-activated protein kinase; PKG, Protein kinase G; PDE5, Phosphodiesterase type 5; ROS, Reactive oxygen species.

PDE5 contributes to the regulation of intracellular cyclic GMP (cGMP) pools (see Figure 1) that have been shown to be decreased along with protein kinase-G (PKG), a downstream effector of cGMP, in variety of different tumors such as breast cancer, colon cancer and human oral squamous cell carcinoma (hOSCC) (Spoto et al., 2003; Zhu and Strada, 2007; Di et al., 2010). PDE5 hydrolyzes the 3′, 5′-phosphodiester bond in the second messenger molecule cGMP to biologically inactive 5′-GMP. There is an incomplete understanding of how PDE5 inhibitors act in cancer, yet there are reports of increased apoptosis in different tumor cell types following treatment with PDE5 inhibitors. Possible mechanisms of these anticancer effects via PDE5 inhibition mediated caspase-dependent apoptosis and cell growth arrest may be linked to concomitant increases in regulation of downstream pathways through increased cGMP-PKG levels and subsequent effects on, (1) activation of c-Jun NH2-terminal kinase (JNK), especially JNK1 pathways via phosphorylation of mitogen-activated protein kinase kinase kinase 1 (MEKK1) (Bender and Beavo, 2006), (2) decreased Wnt/β-catenin expression and down-regulation of cyclin D1 (Thompson et al., 2000; Li et al., 2002; Tinsley et al., 2011), (3) inhibition of extracellular-signal regulated kinases 1/2 (ERK1/2) and alterations in the regulation of p42/p44 mitogen activated-protein kinase (MAPK) and p21 pathways (Hou et al., 2006; Das et al., 2008). Increased PDE5 expression was shown to play a role in tumorigenesis in variety of cancers, such as non-small cell lung cancer, urinary bladder cancer, metastatic breast cancer and development of hOSCC (Piazza et al., 2001; Pusztai et al., 2003; Whitehead et al., 2003). Thus, it is hypothesized that inhibiting PDE5 activity may produce antineoplastic actions. There is a small amount of supporting data. Indeed, PDE5 inhibitors, sildenafil and vardenafil induced caspase-dependent apoptosis of B-cell chronic lymphocytic leukemia cells (Sarfati et al., 2003), whereas cytotoxic and growth suppressive effects in various breast cancer, prostate, and colon cells were seen with non-specific PDE5 inhibitors sulindac sulfone and its analogs (Thompson et al., 2000; Piazza et al., 2001; Whitehead et al., 2003; Tinsley et al., 2011). Interestingly, PDE5 inhibitors were shown to alter the tumor microenvironment by augmenting endogenous antitumor immunity by reducing myeloid-derived suppressor cell function (Serafini et al., 2006).

The accumulating body of evidence suggests that PDE5 inhibitors could interfere with the efflux functions of the ABC transporters, thus sensitizing cancer cells toward cytotoxic agents that are substrates of ABC transporters (Ding et al., 2011; Shi et al., 2011; Chen et al., 2012) (see Figure 1). For example, cGMP is a substrate of ABCC4 and ABCC5 transporters that are involved in reducing its intracellular concentrations. Sildenafil reverses this phenomenon through a dual action, first it inhibits PDE5, and secondly it could block the transport function of ABCC4 and ABCC5, thus increasing the intracellular cGMP concentrations (Jedlitschky et al., 2000; Chen et al., 2001). More recently our group reported that specific PDE5 inhibitors could block the function of ABC transporters at clinically achievable concentrations (Ding et al., 2011; Shi et al., 2011; Chen et al., 2012). We showed that sildenafil could block the efflux functions of ABCB1 and ABCG2 transporters in cancer cells, and thus, significantly reversed the MDR-mediated efflux of substrate anticancer drugs, such as mitoxantrone, paclitaxel, and vinca alkaloids (Shi et al., 2011). Other PDE5 inhibitors, vardenafil and tadalafil were also examined for their effect on ABC transporter-mediated efflux in cancer cells. It was found that vardenafil in a concentration-dependent manner, significantly potentiated the cytotoxicity of anticancer agents that are substrates of ABCB1, but not that of ABCC1 or ABCG2 transporters and this effect was significantly greater than that of tadalafil (Ding et al., 2011). Furthermore, sildenafil and vardenafil enhanced the activity of paclitaxel, docetaxel and vinblastine in the ABCC10-transfected HEK293 cells (Chen et al., 2012). Recently, we found that sildenafil significantly enhanced the sensitivity of specific anticancer drugs in different tumor cell lines and in ABCB1- and ABCG2-bearing MDR mouse models (unpublished data). These novel functions of PDE5 inhibitors might explain why in brain tumor models, doxorubicin and herceptin transport efficacy across the blood-brain tumor barrier was enhanced by the addition of sildenafil and vardenafil (Black et al., 2008). Combination chemotherapy with PDE5 inhibitors was shown to produce reactive oxygen species and led to apoptosis that proved to be beneficial in treatment of broad range of cancers. For example, enhanced tumor suppression and apoptotic activity was seen with a sulindac-docetaxel combination in non-small cell lung cancer orthotopic lung tumor model (Whitehead et al., 2003), and with a sulindac-capecitabine combination in breast cancer (Pusztai et al., 2003), and more recently, with the combination of sildenafil-doxorubicin in *in vivo* models of prostate cancer (Das et al., 2010). We suspect that these combination therapies may have produced enhanced anticancer activity partly due to inhibition of specific ABC transporters, which otherwise reduced intracellular concentration of substrate anticancer agents, but this hypothesis needs to be tested. Furthermore, since the PDE5 inhibitors are substrates of multiple ABC transporters, their individual or overlapping roles (Tiwari et al., 2013) in PDE5 disposition is an open area of research.

In summary, there is evidence that suggests that PDE5 inhibitors may have an anticancer action either by increasing cGMP-PKG and coupled downstream events or by their ability to inhibit ABC transporter—mediated s efflux of anticancer drugs. The safety, high tolerability, and wide availability of PDE5 inhibitors have made this class of drug an attractive tool to investigate their role in cancer chemotherapy. Since a detailed understanding of PDE5 inhibitors as anticancer adjuvants is limited, further studies are warranted to characterize their mechanisms and establish their role.
